# Response Surface Methodology for Ultrasound-Assisted Extraction of Astaxanthin from *Haematococcus pluvialis*

**DOI:** 10.3390/md11051644

**Published:** 2013-05-21

**Authors:** Tang-Bin Zou, Qing Jia, Hua-Wen Li, Chang-Xiu Wang, Hong-Fu Wu

**Affiliations:** 1 Department of Nutrition and Food Hygiene, School of Public Health, Guangdong Medical College, Dongguan 523808, China; E-Mails: jiaqing1029@163.com (Q.J.); chineseli@163.com (H.-W.L.); wxiaomin412@163.com (C.-X.W.); 2 Department of Physiology, School of Basic Medical Sciences, Guangdong Medical College, Dongguan 523808, China; E-Mail: hongfuw@126.com

**Keywords:** ultrasound-assisted extraction, astaxanthin, *Haematococcus pluvialis*, response surface methodology

## Abstract

Astaxanthin is a novel carotenoid nutraceutical occurring in many crustaceans and red yeasts. It has exhibited various biological activities including prevention or amelioration of cardiovascular disease, gastric ulcer, hypertension, and diabetic nephropathy. In this study, ultrasound-assisted extraction was developed for the effective extraction of astaxanthin from *Haematococcus pluvialis*. Some parameters such as extraction solvent, liquid-to-solid ratio, extraction temperature, and extraction time were optimized by single-factor experiment and response surface methodology. The optimal extraction conditions were 48.0% ethanol in ethyl acetate, the liquid-to-solid ratio was 20:1 (mL/g), and extraction for 16.0 min at 41.1 °C under ultrasound irradiation of 200 W. Under optimal conditions, the yield of astaxanthin was 27.58 ± 0.40 mg/g. The results obtained are beneficial for the full utilization of *Haematococcus pluvialis*, which also indicated that ultrasound-assisted extraction is a very useful method for extracting astaxanthin from marine life.

## 1. Introduction

**Figure 1:**
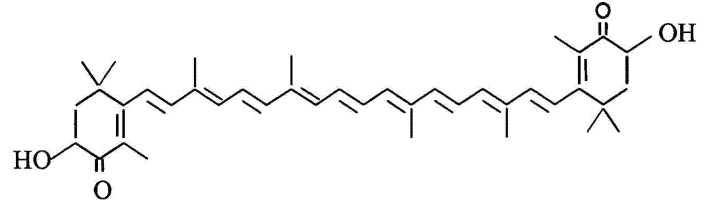
Structure of astaxanthin.

Carotenoids are phytochemicals considered beneficial in the prevention of a variety of major diseases [1,2]. Astaxanthin is one of approximately 700 naturally occurring carotenoids, which are common in crustacean shells, salmon, fish eggs, and asteroideans [3]. Owing to its poor transformation into vitamin A, astaxanthin possesses an antioxidant activity that is approximately 10 times more potent than that of any other carotenoids. This potent antioxidant activity arises from the structural characteristics of astaxanthin. Seen from Figure 1, it is a xanthophyll with hydroxyl and keto endings on each ionone ring, both of which provide a more polar configuration than other carotenoids [4]. Astaxanthin is known to exhibit a wide variety of biological activities including prevention or amelioration of cardiovascular disease, gastric ulcer, hypertension, and diabetic nephropathy [5,6,7,8], most of which are believed to be based on the antioxidant activity inherent to astaxanthin.

The extraction of active ingredients from *Haematococcus pluvialis* can be carried out in various ways, such as maceration extraction, solid-phase microextraction and hydrodistillation [9,10]. Usually, these conventional extraction methods are time- and solvent-consuming. In recent years, various novel extraction methods have been developed for the extraction of active components from plants, such as ultrasonic-assisted extraction (UAE), supercritical fluid extraction, enzymatic extraction, and dispersive liquid-liquid microextraction [11,12,13,14]. Among these, UAE is a rapid and efficient extraction technique. The enhancement in extraction obtained by using ultrasound is mainly attributed to the effect of acoustic cavitations produced in the solvent by the passage of an ultrasound wave [15,16]. Ultrasound also exerts a mechanical effect, allowing greater penetration of solvent into the tissue, increasing the contact surface area between the solid and liquid phase. As a result, the solute quickly diffuses from the solid phase to the solvent [17]. Therefore, UAE has been widely applied to the extraction of many natural products [18,19,20,21]. However, it was unknown whether the extraction efficiency of astaxanthin from *Haematococcus* *pluvialis* could be improved by the UAE. 

Response surface methodology (RSM) was originally described by Box and Wilson as being effective for responses that are influenced by many factors and their interactions [22]. It has been successfully demonstrated that RSM can be used to optimize the total flavonoid compound from many medicinal plants [23]. In the present study, astaxanthin was extracted by UAE and quantified by high-performance liquid chromatography with diode array detection (HPLC-DAD). The effects of several experimental parameters, such as extraction solvent, liquid-to-solid ratio, extraction temperature, and extraction time, on the extraction efficiency of astaxanthin from *Haematococcus pluvialis* were optimized by RSM. The crude extract obtained can be used either in some astaxanthin-related health care products or the further isolation and purification of astaxanthin. Thus, the results will provide valuable information for the full utilization of *Haematococcus pluvialis*.

## 2. Results and Discussion

### 2.1. Chromatographic Results

**Figure 2:**
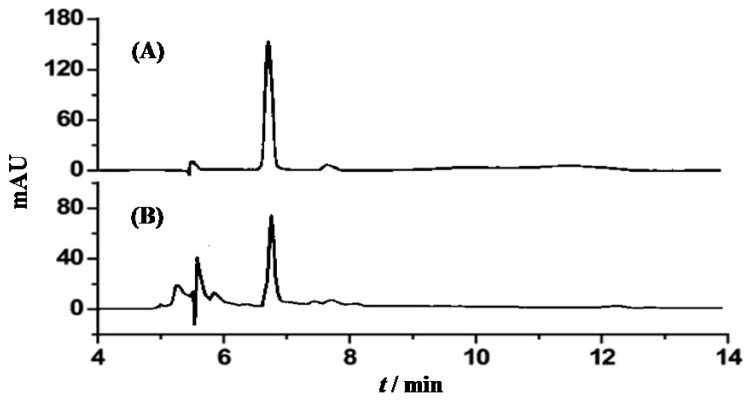
Chromatograms of astaxanthin in standard solution (**A**) and in the sample (**B**).

The chromatograms of astaxanthin in standard solution and in the sample are shown in Figure 2. Astaxanthin in standard solution and in the sample had a retention time of 6.72 min (Figure 2A) and 6.75 min (Figure 2B), respectively. The peak area was used to calculate the amount of astaxanthin from the standard curve. 

### 2.2. Effect of Extraction Solvent on the Astaxanthin Yield

**Figure 3:**
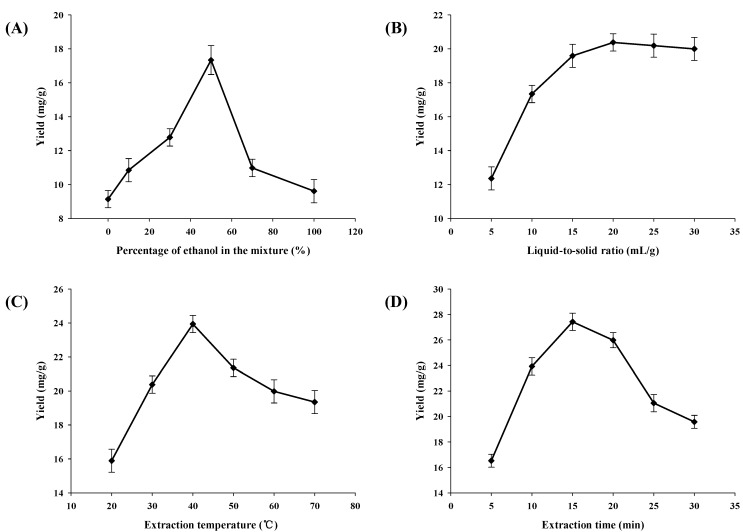
Effects of some parameters on the astaxanthin yield. (**A**) Effect of ethanol concentration on the astaxanthin yield, other conditions were fixed: liquid-to-solid ratio was 10:1, extraction temperature was 30 °C, and extraction for 10 min; (**B**) Effect of liquid-to-solid ratio on the astaxanthin yield, other conditions were fixed: ethanol concentration was 50%, extraction temperature was 30 °C, and extraction for 10 min; (**C**) Effect of extraction temperature on the astaxanthin yield, other conditions were fixed: ethanol concentration was 50%, liquid-to-solid ratio was 20:1, and extraction for 10 min; (**D**) Effect of extraction time on the astaxanthin yield, other conditions were fixed: ethanol concentration was 50%, liquid-to-solid ratio was 20:1, and extraction temperature was 40 °C.

The choice of an extracting solvent was the first crucial step towards parameter optimization, which has a strong impact on the yield of extraction. Different solvents will yield different amount and composition of extract. Therefore, suitable extracting solvent should be selected for the extraction. In this study, a mixture of ethanol and ethyl acetate was employed as extraction solvent [24]. The effect of different proportions of ethanol in the mixture on the yield of astaxanthin was evaluated, and other extraction parameters were constant. The results are shown in Figure 3A, the yields of astaxanthin extracted by pure ethyl acetate and ethanol were at the same level, which were 9.13 ± 0.47 mg/g and 9.61 ± 0.68 mg/g, respectively. When the ethanol concentration increased from 10% to 50%, the yield of astaxanthin significantly increased, followed by a sharp decrease with further increase of ethanol concentration from 50% to 70%. The yield of astaxanthin reached the maximum value at 50% ethanol in ethyl acetate, which was 17.34 ± 0.85 mg/g. The results indicated that 50% ethanol was suitable for the extraction of astaxanthin from *Haematococcus* *pluvialis*. The yield of astaxanthin extracted by 50% ethanol was markedly higher than that extracted by 70% ethanol, which was 10.97 ± 0.52 mg/g. Thus, 50% ethanol in ethyl acetate was used in the subsequent experiments.

### 2.3. Effect of Liquid-to-Solid Ratio on the Astaxanthin Yield

The effect of liquid-to-solid ratio on the astaxanthin yield was investigated, and other extraction parameters were constant. The results are shown in Figure 3B, when the liquid-to-solid ratio increased from 5:1 to 20:1, the yield of astaxanthin increased with the increase of the liquid-to-solid ratio. When the liquid-to-solid ratio increased from 20:1 to 30:1, the yield of astaxanthin almost unchanged with the increase of the liquid-to-solid ratio. The maximum yield obtained was 20.38 ± 0.52 mg/g at 20:1. Generally, the large liquid-to-solid ratio can dissolve constituents more effectively, leading to an enhancement of the extraction yield [25]. However, this will induce the waste of solvent. On the other hand, a small liquid-to-solid ratio will result in a lower extraction yield [26]. Therefore, the choice of a proper solvent volume is significant. In this study, the yield of astaxanthin significantly increased when the liquid-to-solid ratio increased from 5:1 to 20:1. After 20:1, the yield of astaxanthin was almost unchanged. Thus, the liquid-to-solid ratio at 20:1 was used in the subsequent experiments.

### 2.4. Effect of Extraction Temperature on the Astaxanthin Yield

The effect of extraction temperature on the astaxanthin yield was investigated. Temperature is also an important factor in the extraction of heat sensitive compounds. Along with the increase of temperature, the solvent diffusion rate and the mass transfer intensification result in the dissolution of objective components. Meanwhile, the dissolution of impurities can also increase, and some thermal labile components can decompose [27]. In this study, extraction was carried out at different temperatures while other extraction parameters were constant. The results are shown in Figure 3C, the yield of astaxanthin was improved when the extraction temperature increased from 20 to 40 °C, and then the yield decreased from 40 to 70 °C due to the degradation of astaxanthin. The highest yield obtained was 23.94 ± 0.43 mg/g at 40 °C. Similar results were observed in the extraction of anthocyanins from mulberry at high temperature [16]. Thus, 40 °C was used in the subsequent experiments.

### 2.5. Effect of Extraction Time on the Astaxanthin Yield

The effect of extraction time on the astaxanthin yield was investigated, and other extraction parameters were constant. The results are shown in Figure 3D, the yield of astaxanthin increased from 5 to 15 min, and then the yield decreased from 15 to 30 min. The maximum yield obtained was 27.43 ± 0.68 mg/g at 15 min. Generally, time duration can influence the extraction yield [28]. Before the establishment of equilibrium for the objective constituents in and out of plant cells, the extraction yield increases with time. However, it can not increase after the establishment of equilibrium [27]. Thus, 15 min was chosen as optimal extraction time. 

### 2.6. Optimization of the Astaxanthin Yield

The astaxanthin yield was further optimized through the RSM approach. A fixed liquid-to-solid ratio (20:1) was chosen. The coded and actual levels of the three variables in Table 1 were selected to maximize the yield. In total, 17 experiments were designated, from which 12 were factorial experiments and 5 were zero-point tests performed to estimate the errors. 

**Table 1:** Coded and actual levels of three variables.

Independent variables	Coded levels		
	−1	0	1
Ethanol concentration (*X*_1_, %)	30	50	70
Extraction temperature (*X*_2_, °C)	30	40	50
Extraction time (*X*_3_, min)	10	15	20

Table 2 shows the treatments with coded levels and the experimental results of astaxanthin yield in *Haematococcus pluvialis*. The yield ranged from 15.46 to 27.48 mg/g. The maximum yield was recorded under the experimental conditions of *X*_1_ = 48.0%, *X*_2_ = 41.1 °C, and *X*_3_ = 16.0 min. By applying multiple regression analysis to the experimental data, the response variable and the test variables are related by the following second-order polynomial equation:

*Y* = 27.38 ‒ 1.35*X*_1_ + 0.76*X*_2_ + 1.19*X*_3_ + 0.66*X*_1_*X*_2_ +0.35*X*_1_*X*_3_ ‒ 0.19*X*_2_*X*_3_ ‒ 6.12*X*_1_^2^ ‒ 3.00*X*_2_^2^ ‒ 2.71*X*_3_^2^

**Table 2:** Experimental designs using Box-Behnken and results.

Treatment no.	Coded levels			Astaxanthin yield (mg/g)
	*X*_1_	*X*_2_	*X*_3_	
1	−1	0	1	20.87
2	−1	0	−1	19.02
3	0	0	0	27.18
4	1	0	1	18.77
5	0	0	0	27.45
6	0	−1	−1	19.72
7	0	0	0	27.48
8	1	1	0	18.47
9	0	1	1	23.25
10	0	−1	1	22.28
11	1	0	−1	15.52
12	0	0	0	27.41
13	1	−1	0	15.46
14	0	1	−1	21.43
15	−1	−1	0	19.36
16	0	0	0	27.39
17	−1	1	0	19.74

Table 3 shows the analysis of variance (ANOVA) for the regression equation. The linear term and quadratic term were highly significant (*p* < 0.01). The lack of fit was used to verify the adequacy of the model and was not significant (*p* > 0.05), indicating that the model could adequately fit the experiment data. 

**Table 3:** Analysis of variance (ANOVA) for the regression equation.

Source	Sum of squares	Degrees of freedom	Mean square	*F* value	*p* value
Model	281.13	9	31.24	1057.31	<0.0001
*X*_1_	14.50	1	14.50	490.77	<0.0001
*X*_2_	4.61	1	4.61	155.89	<0.0001
*X*_3_	11.23	1	11.23	380.25	<0.0001
*X*_1_*X*_2_	1.73	1	1.73	58.53	0.0001
*X*_1_*X*_3_	0.49	1	0.49	16.59	0.0047
*X*_2_*X*_3_	0.14	1	0.14	4.63	0.0683
*X*_1_^2^	157.95	1	157.95	5346.26	<0.0001
*X*_2_^2^	37.89	1	37.89	1282.46	<0.0001
*X*_3_^2^	30.97	1	30.97	1048.41	<0.0001
Residual	0.21	7	0.030		
Lack of fit	0.15	3	0.050	3.60	0.1239

The adequate precision measures the signal to noise ratio. A ratio greater than 4 is desirable. In this study, the ratio was found to be 90.17, which indicates that this model can be used to navigate the design space. The value of adjusted *R*-squared (0.9983) for the equation is reasonably close to 1, indicated a high degree of correlation between the observed and predicted values, therefore the model is suitable. A very low value of coefficient of the variance (C.V.%) (0.79) clearly indicated a very high degree of precision and reliability of the experimental values. 

**Figure 4:**
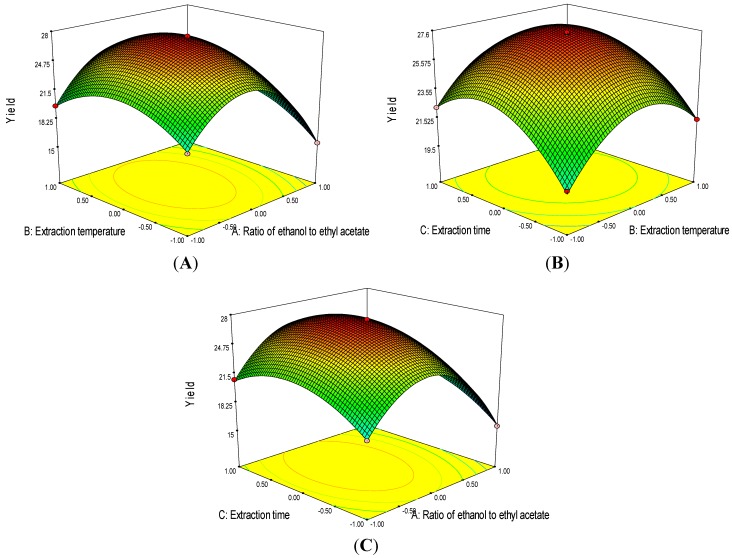
Response surface graphs for the effects of ethanol concentration, extraction temperature, and extraction time on the astaxanthin yield: (**A**) Ethanol concentration (*X*_1_) and extraction temperature (*X*_2_); (**B**) Extraction temperature (*X*_2_) and extraction time (*X*_3_); (**C**) Ethanol concentration (*X*_1_) and extraction time (*X*_3_).

Three-dimensional response surface plots are presented in Figure 4. An increase of ethanol concentration (*X*_1_), extraction temperature (*X*_2_) and extraction time (*X*_3_) result in an initial increase of astaxanthin yield that then decrease when the concentration, temperature and time continue to rise. The optimal values of the selected variables were obtained by solving the regression equation. After calculation by Design Expert software, the optimal extraction conditions of astaxanthin were 48.0% ethanol in ethyl acetate, the liquid-to-solid ratio was 20:1, and extraction for 16.0 min at 41.1 °C, with the corresponding *Y* = 27.61 mg/g. To confirm these results, tests were performed in triplicate under optimized conditions. The astaxanthin yield was 27.58 ± 0.40 mg/g, which clearly showed that the model fitted the experimental data and therefore optimized the astaxanthin extraction procedure from *Haematococcus pluvialis*. 

### 2.7. Comparison of the Results between UAE and Conventional Extraction

The powder of *Haematococcus pluvialis* was extracted by UAE and conventional extraction, respectively. Compared with conventional extraction, UAE was more efficient. Table 4 shows that when samples were extracted for 30 min, the yield of astaxanthin by conventional extraction was only 39% of that by UAE. Given more time in the conventional extraction, such as 120 min, astaxanthin yield was just about 65% of that by UAE. Thus, UAE is a more efficient extraction method. 

**Table 4:** The comparison of ultrasound-assisted extraction (UAE) and conventional extraction.

	UAE	Conventional extraction			
		30 min	60 min	90 min	120 min
**Yield (mg/g)**	27.58 ± 0.40	10.83 ± 0.71	14.72 ± 0.94	16.48 ± 0.67	17.85 ± 0.52

## 3. Experimental Section

### 3.1. Chemicals and Reagents

Astaxanthin standard (purity ≥98%) was purchased from Sigma-Aldrich (St. Louis, MO, USA) and stored at −80 °C. Methanol, ethanol, acetonitrile, dichloromethane, and ethyl acetate were HPLC grade and bought from Fisher Scientific (Fairlawn, NJ, USA). Deionized water was obtained by a purification system and filtrated through a 0.45 μm millipore filter (Pall Life Sciences, Ann Arbor, MI, USA). 

### 3.2. Plant Material

*Haematococcus pluvialis* was obtained from Jingzhou natural Astaxanthin Inc. (Hubei, China), and stored at −80 °C to avoid degradation of thermal compounds. 

### 3.3. Ultrasound-Assisted Extraction

The ultrasound-assisted extraction (UAE) was carried out in an ultrasonic device (KJ1004B, Kejin Instrument Company, Guangzhou, China) with an ultrasound power of 200 W and frequency of 40 kHz, equipped with a digital timer and a temperature controller.

The powder of *Haematococcus pluvialis* (1.0 g) was accurately weighed, and placed in a capped tube, then mixed with an appropriate amount of extraction solution. After wetting the material, the tube with suspension was immersed into water in the ultrasonic device, and irradiated for the predetermined extraction time. After ultrasonic extraction, the sample was centrifuged at 8500 rpm for 10 min, and the supernatant was collected. The precipitation was taken back and extracted again under the same conditions. The extracts of the twice-extraction were mixed and filtered using a 0.45 μm syringe filter (Pall Life Sciences, Ann Arbor, MI, USA) for HPLC analysis. 

### 3.4. Experimental Design

The extraction parameters were optimized using response surface methodology (RSM) [29]. A Box-Behnken experiment was employed in this regard. Ethanol concentration (*X*_1_), extraction temperature (*X*_2_), and extraction time (*X*_3_) were chosen for independent variables. The range and center point values of the three independent variables presented in Table 1 are based on the results of preliminary single factor experiments. The experimental design consists of 12 factorial experiments and 5 replicates of the central point. Astaxanthin yield was selected as the responses for the combination of the independent variables given in Table 2. Experimental runs were randomized, to minimize the effects of unexpected variability in the observed responses. The variables were coded according to the following equation:

*x* = (*X_i_* ‒ *X*_0_)/Δ*X*

where *x* is the coded value, *X_i_* is the corresponding actual value, *X*_0_ is the actual value in the center of the domain, and Δ*X* is the increment of *X_i_* corresponding to a variation of 1 unit of *x*. The mathematical model corresponding to the Box-Behnken design is:

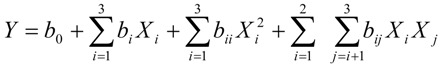

where *Y* is the predicted response, *b*_0_ is the model constant, *b_i_*, *b_ii_* and *b_ij_* are the model coefficients. They represent the linear, quadratic and interaction effects of the variables. Analysis of the experimental design data and calculation of predicted responses were carried out using Design Expert software (Version 7.1.6, Stat-Ease, Inc., Minneapolis, MN, USA). Additional confirmation experiments were subsequently conducted to verify the validity of the statistical experimental design.

### 3.5. Conventional Extraction

The powder of *Haematococcus pluvialis* (1.0 g) was suspended in 20 mL of 50% ethanol in ethyl acetate. After wetting the material, conventional extraction was carried out at room temperature for 30, 60, 90 and 120 min, respectively. After the extraction, the astaxanthin extracts were treated the same as UAE. 

### 3.6. HPLC Analysis

Astaxanthin was analyzed by a Waters (Milford, MA, USA) e2695 separations module with a Waters 2998 diode array detector. An elite^®^ C18 column (250 mm × 4.6 mm, 5 μm) was used. The mobile phase consisted of a mixture of water:methanol:dichloromethane:acetonitrile (4.5:28.0:22.0:45.5, v/v/v/v) at a flow rate of 1.0 mL/min [30]. The wavelength of detection was 476 nm, column temperature was 25 °C, injection volume was 20 μL. Astaxanthin was quantified based on peak area and comparison with the standard curve. 

### 3.7. Statistical Analysis

Experiments were performed in triplicate and data were expressed as the mean ± standard deviation. Analysis of the experimental design data and calculation of predicted responses were carried out by Design Expert software. Differences were considered significant if *p* < 0.05. 

## 4. Conclusions

In the present study, ultrasound-assisted extraction has been developed for the extraction of astaxanthin from *Haematococcus pluvialis*. Ultrasonic wave is a powerful tool, which can efficiently improve the extracting performance of astaxanthin. The RSM was successfully employed to optimize the extraction and several experimental parameters have been evaluated. The results showed that extraction solvent, extraction temperature, and extraction time all had significant effects on the yield of astaxanthin. The best combination of response function was 48.0% ethanol in ethyl acetate, the liquid-to-solid ratio was 20:1, and extraction for 16.0 min at 41.1 °C under ultrasound irradiation of 200 W. Under optimal conditions, the yield of astaxanthin was 27.58 ± 0.40 mg/g. The results obtained are beneficial for the full utilization of *Haematococcus pluvialis*, which also indicated that UAE is a powerful tool for extracting astaxanthin from marine life.

## References

[1] Riccioni G., D’Orazio N., Franceschelli S., Speranza L. (2011). Marine carotenoids and cardiovascular risk markers. Mar. Drugs.

[2] Wolf A.M., Asoh S., Hiranuma H., Ohsawa I., Iio K., Satou A., Ishikura M., Ohta S. (2010). Astaxanthin protects mitochondrial redox state and functional integrity against oxidative stress. J. Nutr. Biochem..

[3] Inoue M., Tanabe H., Matsumoto A., Takagi M., Umegaki K., Amagaya S., Takahashi J. (2012). Astaxanthin functions differently as a selective peroxisome proliferator-activated receptor gamma modulator in adipocytes and macrophages. Biochem. Pharmacol..

[4] Pashkow F.J., Watumull D.G., Campbell C.L. (2008). Astaxanthin: A novel potential treatment for oxidative stress and inflammation in cardiovascular disease. Am. J. Cardiol..

[5] Fassett R.G., Coombes J.S. (2011). Astaxanthin: A potential therapeutic agent in cardiovascular disease. Mar. Drugs.

[6] Kim J.H., Kim Y.S., Song G.G., Park J.J., Chang H.I. (2005). Protective effect of astaxanthin on naproxen-induced gastric antral ulceration in rats. Eur. J. Pharmacol..

[7] Hussein G., Nakamura M., Zhao Q., Iguchi T., Goto H., Sankawa U., Watanabe H. (2005). Antihypertensive and neuroprotective effects of astaxanthin in experimental animals. Biol. Pharm. Bull..

[8] Naito Y., Uchiyama K., Aoi W., Hasegawa G., Nakamura N., Yoshida N., Maoka T., Takahashi J., Yoshikawa T. (2004). Prevention of diabetic nephropathy by treatment with astaxanthin in diabetic db/db mice. Biofactors.

[9] Sultana B., Hussain Z., Asif M., Munir A. (2012). Investigation on the antioxidant activity of leaves, peels, stems bark, and kernel of mango (*Mangifera indica* L.). J. Food Sci..

[10] Gebara S.S., de Oliveira Ferreira W., Re-Poppi N., Simionatto E., Carasek E. (2011). Volatile compounds of leaves and fruits of *Mangifera indica* var. coquinho (Anacardiaceae) obtained using solid phase microextraction and hydrodistillation. Food Chem..

[11] Vinatoru M. (2001). An overview of the ultrasonically assisted extraction of bioactive principles from herbs. Ultrason. Sonochem..

[12] Yang H., Li X., Tang Y., Zhang N., Chen J., Cai B. (2009). Supercritical fluid CO_2_ extraction and simultaneous determination of eight annonaceous acetogenins in *Annona* genus plant seeds by HPLC-DAD method. J. Pharm. Biomed. Anal..

[13] Hardlei T.F., Morkbak A.L., Nexo E. (2007). Enzymatic extraction of cobalamin from monoclonal antibody captured haptocorrin and transcobalamin. Clin. Biochem..

[14] Campillo N., Vinas P., Ferez-Melgarejo G., Hernandez-Cordoba M. (2013). Dispersive liquid-liquid microextraction for the determination of macrocyclic lactones in milk by liquid chromatography with diode array detection and atmospheric pressure chemical ionization ion-trap tandem mass spectrometry. J. Chromatogr. A.

[15] Ghafoor K., Choi Y.H., Jeon J.Y., Jo I.H. (2009). Optimization of ultrasound-assisted extraction of phenolic compounds, antioxidants, and anthocyanins from grape (*Vitis vinifera*) seeds. J. Agric. Food Chem..

[16] Zou T.B., Wang M., Gan R.Y., Ling W.H. (2011). Optimization of ultrasound-assisted extraction of anthocyanins from mulberry, using response surface methodology. Int. J. Mol. Sci..

[17] Rostagno M.A., Palma M., Barroso C.G. (2003). Ultrasound-assisted extraction of soy isoflavones. J. Chromatogr. A.

[18] Da Porto C., Porretto E., Decorti D. (2013). Comparison of ultrasound-assisted extraction with conventional extraction methods of oil and polyphenols from grape (*Vitis vinifera* L.) seeds. Ultrason. Sonochem..

[19] Wang X.S., Wu Y.F., Dai S.L., Chen R., Shao Y. (2012). Ultrasound-assisted extraction of geniposide from *Gardenia jasminoides*. Ultrason. Sonochem..

[20] Hossain M.B., Brunton N.P., Patras A., Tiwari B., O’Donnell C.P., Martin-Diana A.B., Barry-Ryan C. (2012). Optimization of ultrasound assisted extraction of antioxidant compounds from marjoram (*Origanum majorana* L.) using response surface methodology. Ultrason. Sonochem..

[21] Xia E.Q., Yu Y.Y., Xu X.R., Deng G.F., Guo Y.J., Li H.B. (2012). Ultrasound-assisted extraction of oleanolic acid and ursolic acid from *Ligustrum lucidum* Ait. Ultrason. Sonochem..

[22] Box G., Wilson K. (1951). On the experimental attainment of optimum conditions. J. R. Stat. Soc..

[23] Liu W., Yu Y., Yang R., Wan C., Xu B., Cao S. (2010). Optimization of total flavonoid compound extraction from *Gynura medica* leaf using response surface methodology and chemical composition analysis. Int. J. Mol. Sci..

[24] Sarada R., Vidhyavathi R., Usha D., Ravishankar G.A. (2006). An efficient method for extraction of astaxanthin from green alga *Haematococcus pluvialis*. J. Agric. Food Chem..

[25] Li H., Chen B., Yao S. (2005). Application of ultrasonic technique for extracting chlorogenic acid from *Eucommia ulmodies* Oliv. (*E. ulmodies*). Ultrason. Sonochem..

[26] Valachovic P., Pechova A., Mason T.J. (2001). Towards the industrial production of medicinal tincture by ultrasound assisted extraction. Ultrason. Sonochem..

[27] Dong J., Liu Y., Liang Z., Wang W. (2010). Investigation on ultrasound-assisted extraction of salvianolic acid B from *Salvia miltiorrhiza* root. Ultrason. Sonochem..

[28] Galhiane M.S., Rissato S.R., Chierice G.O., Almeida M.V., Silva L.C. (2006). Influence of different extraction methods on the yield and linalool content of the extracts of *Eugenia uniflora* L.. Talanta.

[29] Bezerra M.A., Santelli R.E., Oliveira E.P., Villar L.S., Escaleira L.A. (2008). Response surface methodology (RSM) as a tool for optimization in analytical chemistry. Talanta.

[30] Lopez-Cervantes J., Sanchez-Machado D.I., Gutierrez-Coronado M.A., Rios-Vazquez N.J. (2006). Quantification of astaxanthin in shrimp waste hydrolysate by HPLC. Biomed. Chromatogr..

